# Intestinal Parasitic Infections in HIV-Infected Patients, Lao People’s Democratic Republic

**DOI:** 10.1371/journal.pone.0091452

**Published:** 2014-03-24

**Authors:** Phimpha Paboriboune, Niranh Phoumindr, Elisabeth Borel, Khamphang Sourinphoumy, Saykham Phaxayaseng, Elodie Luangkhot, Bouachanh Sengphilom, Yathmany Vansilalom, Peter Odermatt, Eric Delaporte, Jean- François Etard, Meja Rabodonirina

**Affiliations:** 1 Centre d’Infectiologie Christophe Mérieux du Laos, Ministry of Health, Vientiane, Lao People’s Democratic Republic; 2 UMI 233, Institut de Recherche pour le Développement – Université Montpellier 1, Montpellier, France; 3 Department of Parasitology, University of Health Sciences, Vientiane, Lao People’s Democratic Republic; 4 Faculté de Médecine Lyon-Sud Charles Mérieux, Université Lyon 1, Lyon, France; 5 Department of Infectious Diseases, Provincial Hospital, Savannakhet, Lao People’s Democratic Republic; 6 Department of Infectious Diseases, Setthatirath Hospital, Vientiane, Lao People’s Democratic Republic; 7 Service de Parasitologie, Hospices Civils de Lyon, Lyon, France; 8 Department of Epidemiology and Public Health, Swiss Tropical and Public Health Institute, Basel, Switzerland; Institute of Infection and Global Health, United Kingdom

## Abstract

**Background:**

HIV infection is an emerging problem in Laos. We conducted the first prospective study on intestinal parasites, including opportunistic protozoa, in newly diagnosed HIV infected patients, with or without diarrhea. The aims were to describe the spectrum of infections, to determine their prevalence and to assess their associations with diarrhea, CD4 cell count, place of residence and living conditions.

**Methodology:**

One to three stool samples over consecutive days were obtained from 137 patients. The Kato thick smear method, formalin-ethyl concentration and specific stains for coccidia and microsporidia diagnosis were performed on 260 stool samples. Baseline characteristics regarding relevant demographics, place of residence and living conditions, clinical features including diarrhea, were collected using a standardized questionnaire.

**Principal Findings:**

The 137 patients were young (median age: 36 years) and severely immunocompromised (83.9% at WHO stage 3 or 4, median CD4 cell count: 41/mm^3^). Diarrhea was present in 43.0% of patients. Parasite infection was found in 78.8% of patients, infection with at least two species in 49.6%. Prevalence rates of protozoan and helminth infections were similar (54.7% and 58.4% respectively). *Blastocystis* sp. was the most frequent protozoa (26.3%). *Cryptosporidium* sp., *Cytoisospora belli* and microsporidia, found at low prevalence rates (6.6%, 4.4%, 2.9%, respectively), were described for the first time in Laos. *Cryptosporidium* sp. was associated with persistent diarrhea. *Strongyloides stercoralis* was the most prevalent helminth following *Opisthorchis viverrini* (20.4% and 47.5% respectively). The most immunocompromised patients, as assessed by a CD4 count ≤ 50 cells/mm^3^, were more likely to be infected with intestinal parasites.

**Conclusions/Significance:**

HIV infection was mainly diagnosed at an advanced stage of immunosuppression in Lao patients. Intestinal parasite infections were highly prevalent regardless of their diarrheal status. Opportunistic infections were reported. Improving the laboratory diagnosis of intestinal parasite infections and the knowledge on their local risk factors is warranted.

## Introduction

Diarrhea is a major cause of morbidity in HIV-infected patients. Moreover, it is recognized as an independent marker of poor prognosis [1]. In previous studies from industrialized countries, infectious causes were demonstrated to be prevalent among the etiologies of diarrhea, especially parasites [1]. There is a paucity of microbiological data from emerging and developing countries because of the lack of equipment, reagents and/or trained laboratory staff [2]. Nevertheless, the identification and characterization of infectious agents are important at two levels: (i) for patient management by confirming or ruling out a clinical diagnosis, thereby allowing an appropriate treatment, (ii) for public healthcare policy by determining the true prevalence of a pathogen in a country or region and yielding epidemiological risk factors for specific infections.

Lao People’s Democratic Republic (Lao PDR, Laos) is a landlocked country in the Mekong region of Southeast Asia, surrounded by countries with high HIV prevalence such as Thailand. The first case of HIV infection was identified in Laos in 1990 and antiretroviral therapy (ART) was provided for the first time in 2003 in the Savannakhet Regional Hospital by Médecins Sans Frontières [3]. Laos is considered to be a low-level HIV epidemic country with an estimated prevalence of 0.3% and an estimated 12,000 people living with HIV/AIDS in 2012 [4]. The development of the National AIDS Response led in 2012 to the treatment with ART of 2,212 patients in seven centers [4].

Parasitological analysis in Lao hospital laboratories is performed using direct examination of fresh unconcentrated stools and the Kato thick smear technique [5], allowing the diagnosis of helminth infections which are frequent in the general population of Laos [6], [7], [8], [9], [10], [11]. Little information is available on intestinal protozoan infections. *Blastocystis*, an emerging pathogen, was recently reported for the first time in the general population [9]. Regarding opportunistic infections, information on major intestinal parasite infections among HIV patients such as *Cryptosporidium* and microsporidia has not been reported to date due to rare diagnostic performance.

To fill this gap, we conducted a cross-sectional study in two referral hospitals for HIV/AIDS care. The purpose was to assess the diversity and prevalence of intestinal parasitic infections in antiretroviral-naïve HIV-infected patients, and to determine their association with diarrhea, CD4 cell counts, place of residence and living conditions.

## Methods

### Ethics statement

The study protocol, including the consent procedure, was reviewed and approved by the Ethical Committee of the University of Health Sciences (Ministry of Public Health, Laos, reference no. 009/08). Patients were included in the study on a voluntary basis. After an information sheet had been read aloud, a written consent form was signed and obtained from the patients (or parents in the case of minors less than 16 years of age). This research followed the principles expressed in the Declaration of Helsinki. All infections diagnosed were treated using a standard treatment protocol.

### Study sites and population

The study was carried out from October 2009 to September 2010 in two public hospitals of Laos: Setthathirath Hospital in Vientiane (Vientiane Capital) and Savannakhet Regional Hospital (Savannakhet province, Southern part of Laos). These hospitals are two of the seven HIV care and treatment centers of the country. Patients admitted for the care of a newly diagnosed HIV-infection were included before any treatment, including ART, was started. Each patient underwent a standard clinical assessment and a questionnaire was completed. The CD4 cell count was assessed and up to three stool samples were collected and analyzed.

### Data collection

Baseline characteristics regarding relevant demographics, place of residence and living conditions (province of Laos, urban/rural housing, access to drinking water and in-house toilets, animal contact), clinical features (fever, weight loss, anorexia, diarrhea, nausea, vomiting, abdominal pain, and pulmonary, neurological and mucocutaneous symptoms) were collected at inclusion by the physician using a standardized questionnaire. According to World Health Organization definitions, diarrhea was defined as the passage of three or more loose or liquid stools per day [12]. It was considered acute when it lasted less than two weeks and persistent when it lasted 14 days or longer [12]. The immunological status of the patients related to HIV disease was assessed using the 2007 - revised World Health Organization HIV clinical staging and disease classification system [13].

### Laboratory examination

Serological diagnosis of HIV infection was made using two rapid screening tests: Determine HIV 1/2 (Inverness Medical, Tokyo, Japan) and Uni-Gold HIV (Trinity Biotech, Bray, Ireland). In the case of discordant results, the diagnosis was confirmed by the ELISA test Vironostika HIV Uniform II plus O (BioMérieux, Marcy-l’Etoile, France). HIV viral load testing was not prescribed to patients with a newly diagnosed HIV infection because of limited availability of the technique. The CD4 lymphocyte count was determined by flow cytometry (Cyflow SL and Cyflow Counter, Partec GmbH, Munster, Germany).

Laboratory investigations for opportunistic infections were limited to direct diagnosis of mycobacterial infections and cryptococcosis using Ziehl-Neelsen stain on sputum samples and India ink test or antigen detection in cerebrospinal fluid samples, respectively.

### Stool sample analysis

All patients were asked to provide three stool samples from three consecutive days. Each fresh stool specimen was processed as follows: the technicians of the hospital laboratories performed direct microscopic examination and examination of a Kato thick smear [5]. The sample was then preserved in 10% formalin and forwarded to the parasitological department of the Faculty of Medicine, University of Heath Sciences (Vientiane, Laos). The formalin-preserved samples were subjected to two techniques: formalin-ethyl concentration technique for protozoa and helminths diagnosis [14] and modified acid-fast staining for coccidia diagnosis (*Cryptosporidium* spp., *Cyclospora cayetanensis*, *Cytoisospora belli*, *Sarcocystis hominis*) [15]. The diagnoses were made with the assistance of parasitologists from the University of Lyon (France).

Two smears from formalin-preserved samples were also prepared for microsporidia diagnosis. They were sent to the University of Lyon where the diagnosis was made using the Weber trichrome stain [16] and the fluorescent stain with Uvitex 2B (Uvibio, LDBio Diagnostics, Lyon, France) [17].

Patients were considered infected with a parasite species if at least one of their stool samples was positive.

### Statistical analysis

EpiData freeware, version 3.1 (EpiData Association, Odense, Denmark) was used for data entry. Analyses were performed using STATA software, version 11 (StataCorp., College Station, Texas, USA). Descriptive statistics were carried out. Frequencies were calculated for categorical variables. Proportions were compared using Fisher’s exact test. Odds ratio (OR) and 95% confidence intervals (CI) were calculated by using a bi-variable logistic regression. Tests were considered significant at *P* value≤0.05. Factors potentially associated with intestinal parasite infections were diarrhea, CD4 cell count, place of residence and living conditions.

## Results

### Study population

A total of 140 HIV-positive patients met the criteria for inclusion in the study and gave their consent. Amongst them, 137 patients had complete data records (the CD4 cell count lacked for three patients) and were included in the final analysis. The median age was 36 years (interquartile range [IQR]: 28–41). Males accounted for 53.5% of patients. Sixty seven patients (48.9%) came from the Northern provinces: Luang Namtha, Luang Prabang, Xaiyabury, Vientiane capital, Vientiane province, Xieng Kouang. The others came from the Southern provinces: Bolikhamxay, Savannakhet, Khammuane, Champasack, Saravane. Seventy four patients (54.0%) lived in rural areas. The proportion of patients living in rural areas was higher in Southern provinces than in Northern provinces (53 (75.7%) vs. 21 (31.3%), OR = 6.8, 95% CI = 2.9–15.8). One hundred patients (73.0%) had access to clean drinking water, 103 (75.2%) had in-house toilets and 48 (35.0%) reported animal contact, namely poultry (31.4%), pigs (18.2%) and bovine species (16.8%).

The patients were severely immunocompromised, as assessed by WHO clinical staging criteria (115 patients (83.9%) at stage 3 or 4) and by CD4 cell count (median: 41 cells/mm^3^, [IQR]: 14–94 cells/mm^3^). The proportion of patients at the advanced stages 3 or 4 was significantly higher in Southern provinces than in Northern provinces: 67 (95.7%) vs. 48 (71.6%), OR = 8.8, 95% CI = 2.3–34.1). Laboratory diagnosis of tuberculosis was performed in 49 patients (35.8%), leading to the identification of 10 cases of pulmonary tuberculosis (7.3% of all the patients). Eleven cases of cryptococcal meningitis (8.0% of all the patients) were diagnosed in the 44 patients (32.1%) who underwent laboratory investigations for cryptococcosis.

Fifty nine patients (43.0%) presented with diarrhea, of whom 20 (14.6%) had persistent diarrhea. The median duration of the diarrheal episodes was 14 days ([IQR]: 5–30). The median number of stools per day was four ([IQR]: 4–5). Table 1 summarizes demographic, clinical and laboratory characteristics of the patients, with or without diarrhea. Compared to patients without diarrhea, patients with diarrhea presented more often with other symptoms: fever (OR = 4.2, 95% CI = 1.5–11.4), anorexia (OR = 2.7, 95% CI = 1.1–7.1), reported weight loss (OR = 2.6, 95% CI = 1.1–6.3), abdominal pain (OR = 5.3, 95% CI = 2.3–12.0), oral candidiasis (OR = 2.2, 95% CI = 1.1–4.5). Diarrheal patients were also diagnosed at a more advanced stage of HIV infection (stages 3–4 *vs*. stages 1–2: OR = 4.1, 95% CI = 1.3–13.4). No demographic characteristic or a CD4 cell count ≤ 50/mm^3^ was found to be associated with diarrhea. Amongst environmental conditions, living in the Southern provinces was associated with diarrhea compared to living in the Northern provinces (36 (61.0%) vs. 23 (39.0%), OR = 2.0, 95% CI = 1.0–4.2).

**Table 1:** Demographic, clinical and laboratory characteristics of HIV-infected patients with regard to diarrhea, Laos (n = 137).

	Total (%)	Diarrhea, n = 59 (%)	OR (95% CI)
Demographic features			
***Age group (years)***			
< 35 y	77 (56.2)	34 (57.3)	1.1 (0.6–2.2)
≥ 35 y	60 (43.8)	25 (42.4)	1.00
*Sex*			
Male	73 (53.3)	35 (59.3)	1.5 (0.8–3.1)
Female	64 (46.7)	24 (40.7)	1.00
**Clinical features**			
***Fever***			
Yes	106 (77.4)	53 (89.8)	4.2 (1.5–11.4)
No	31 (22.6)	6 (10.2)	1.00
***Anorexia***			
Yes	109 (79.6)	52 (88.1)	2.7 (1.1–7.1)
No	28 (20.4)	77 (11.9)	1.00
***Reported weight loss***			
Yes	103 (75.2)	50 (84.8)	2.6 (1.1–6.3)
No	34 (24.8)	9 (15.2)	1.00
***Abdominal pain***			
Yes	50 (36.5)	34 (57.6)	5.3 (2.3–12.0)
No	87 (63.5)	25 (42.4)	1.00
***Nausea and/or vomiting***			
Yes	30 (21.9)	12 (20.3)	0.9 (0.4–1.9)
No	107 (78.1)	47 (79.7)	1.00
***Oral candidiasis***			
Yes	53 (38.7)	29 (49.2)	2.2 (1.1–4.5)
No	84 (61.3)	30 (50.8)	1.00
***Pulmonary signs***			
Yes	60 (43.8)	30 (50.9)	1.7 (0.8–3.3)
No	77 (56.2)	29 (49.1)	1.00
***Neurological signs***			
Yes	18 (13.1)	66 (10.2)	0.6 (0.2–1.8)
No	119 (86.9)	53 (89.8)	1.00
**WHO clinical stages**			
Stage 3–4	115 (83.9)	55 (93.2)	4.1 (1.3–13.4)
Stage 1–2	22 (16.1)	44 (6.8)	1.00
**Laboratory features**			
***CD4 (cells/mm^3^)***			
≤ 50	80 (58.4)	34 (57.6)	0.9 (0.5–1.9)
> 50	57 (41.6)	25 (42.4)	1.00

OR, Odds Ratio.

CI, confidence interval.

### Intestinal parasitic infections

Two hundred and sixty stool specimens were obtained and analyzed. Thirty two patients (23.3%) gave three stool samples, 59 (43.1%) gave two samples and 46 (33.6%) gave one sample. Table 2 presents the parasite species diagnosed with regard to the region of residence. At least one parasite species was identified in the majority of patients (78.8%), two or more species in 68 patients (49.6%). Seventy five patients (54.7%) were infected with at least one protozoan species. *Blastocystis* spp., diagnosed in 36 patients (26.3%), was the most common protozoa, followed by *Entamoeba histolytica/dispar* (12.4%) and *Giardia intestinalis* (8.8%). Classical opportunistic agents (*Cryptosporidium* spp., *Cytoisospora belli, Cyclospora cayetanensis* and microsporidia) were present, but were less frequent (6.6%, 4.4%, 2.2% and 2.9% respectively). Eighty patients (58.4%) were infected with at least one helminth species. The most common helminths were *Opisthorchis viverrini* and *Strongyloides stercoralis*, detected in 65 (47.5%) and 28 patients (20.4%) respectively. No *Schistosoma mekongi* infection or cestode infections were diagnosed. The prevalence rates of helminthiases generally and of strongyloidiasis in particular, were higher in Southern provinces than in Northern provinces (67.1% vs. 49.2%, *P* = 0.03, and 28.5% vs. 11.9%, *P* = 0.01%, respectively). Environmental factors associated with infection with some parasite species were identified: helminth infections were more frequent in individuals who did not have in-house toilets compared to patients who had in-house toilets (79.4% vs. 51.5%, *P* <0.01) and *S. stercoralis* was more frequent in patients living in rural areas compared to patients living in cities (28.4% vs. 11.1%, *P* = 0.01).

**Table 2:** Intestinal parasitic infections in HIV-infected patients by region of residence (Southern or Northern provinces), Laos (n = 137).

	Total (%)	South, n = 70 (%)	North, n = 67 (%)	*P-*value
Parasites				
Any parasite	108 (78.8)	57 (81.4)	51 (76.1)	0.44
At least 2 parasite species	68 (49.6)	39 (55.7)	29 (43.3)	0.14
Any protozoa	75 (54.7)	35 (50.0)	40 (59.7)	0.25
*Cryptosporidium* spp.	9 (6.6)	2 (2.9)	7 (10.5)	0.09
*Cytoisospora belli*	6 (4.4)	3 (4.3)	3 (4.5)	0.95
*Cyclospora cayetanensis*	3 (2.2)	1 (1.4)	2 (3.0)	0.61
*Giardia intestinalis*	12 (8.8)	8 (11.4)	4 (6.0)	0.36
*Entamoeba histolytica/dispar*	17 (12.4)	11 (15.7)	6 (9.0)	0.30
*Entamoeba coli*	1 (0.7)	0	1 (1.5)	n.a.
*Entamoeba hartmanii*	5 (3.7)	1 (1.4)	4 (6.0)	0.20
*Blastocystis* spp.	36 (26.3)	16 (22.9)	20 (29.9)	0.35
Microsporidia	4 (2.9)	2 (2.9)	2 (3.0)	0.96
Any helminth	80 (58.4)	47 (67.1)	33 (49.2)	0.03
*Opisthorchis viverrini*	65 (47.5)	37 (52.9)	28 (41.8)	0.19
Large trematode	1 (0.7)	1 (1.4)	0	n.a.
*Strongyloides stercoralis*	28 (20.4)	20 (28.5)	8 (11.9)	0.01
Hookworm	19 (13.9)	10 (14.3)	9 (13.4)	0.88
*Trichuris trichiura*	3 (2.2)	2 (2.9)	1 (1.5)	0.58
*Ascaris lumbricoides*	1 (0.7)	0	1 (1.5)	n.a.

*P*-value of Fisher exact test.

n.a., not applicable.

Table 3 shows the results from analyses regarding association between intestinal parasites and acute or persistent diarrhea. Compared to acute diarrhea, persistent diarrhea was significantly associated with *Cryptosporidium* sp. infection (20.0% vs. 2.5%, *P* = 0.04).

**Table 3:** Association of intestinal parasites with acute and persistent diarrhea in HIV-infected patients, Laos (n = 59).

	Diarrhea (%)	Acute diarrhea, n = 39 (%)	Persistent diarrhea, n = 20 (%)	*P-*value
Parasites				
Any parasite	44 (74.6)	28 (71.8)	16 (80.0)	0.55
At least 2 parasite species	29 (49.1)	16 (41.0)	13 (65.0)	0.08
Any protozoa	31 (52.5)	18 (46.2)	13 (65.0)	0.27
*Cryptosporidium* spp.	5 (8.5)	1 (2.6)	4 (20.0)	0.04
*Cytoisospora belli*	3 (5.1)	2 (5.1)	1 (5.0)	1.00
*Cyclospora cayetanensis*	1 (1.7)	0	1 (5.0)	n.a.
*Giardia intestinalis*	5 (8.5)	4 (10.3)	1 (5.0)	0.65
*Blastocystis* spp.	19 (32.2)	10 (25.6)	9 (45.0)	0.15
Microsporidia	1 (1.7)	0	1 (5.0)	n.a.
Any helminth	32 (54.2)	21 (53.9)	11 (55.0)	1.00
*Strongyloides stercoralis*	9 (15.3)	6 (15.4)	3 (15.0)	1.00

*P*-value of Fisher exact test.

n.a., not applicable.

Table 4 summarizes significant associations between different intestinal parasite species and CD4 cell count. CD4 count ≤ 50 cells/mm^3^ showed significant positive associations with infection with any parasite (OR = 3.5, 95% CI = 1.4–8.6), with three parasite species (OR = 2.9, 95% CI = 1.1–7.5), with any protozoa (OR = 2.4, 95% CI = 1.2–4.9), with any helminth (OR = 2.0, 95% CI = 1.0–3.9) and with O. *viverrini* (OR = 2.1, 95% CI = 1.0–4.3).

**Table 4:** Association of intestinal parasites with CD4 cell count in HIV-infected patients, Laos (n = 137).

	Total (%)	CD4 ≤50/mm^3^, n = 80 (%)	OR (95% CI)
Parasites			
Any parasite	108 (78.3)	70 (87.5)	3.5 (1.4–8.6)
Three parasite species	30 (21.9)	23 (28.7)	2.9 (1.1–7.5)
Any protozoa	75 (54.7)	51 (63.8)	2.4 (1.2–4.9)
*Cryptosporidium* spp.	9 (6.6)	8 (10.0)	6.2 (0.7–53.2)
*Cytoisospora belli*	6 (4.4)	3 (3.8)	0.7 (0.1–3.6)
*Cyclospora cayetanensis*	3 (2.2)	1 (1.3)	0.3 (0.03–4.0)
*Giardia intestinalis*	12 (8.8)	9 (11.3)	2.3 (0.6–9.0)
*Blastocystis* spp.	36 (26.3)	22 (27.5)	1.2 (0.5–2.5)
Microsporidia	4 (2.9)	3 (3.8)	2.0 (0.2–21.8)
Any helminth	80 (58.4)	52 (65.0)	1.9 (1.0–3.9)
*Strongyloides stercoralis*	28 (20.4)	18 (22.5)	1.4 (0.6–3.2)
*Opisthorchis viverrini*	65 (47.5)	44 (55.0)	2.1 (1.0–4.3)

OR, Odds ratio.

CI, Confidence interval.

## Discussion

Although the HIV prevalence in Laos is estimated to be at the low level of 0. 3%, the absolute number of people living with HIV/AIDS is increasing every year: the estimated number of 3,300 in 2001 reached 12,000 in 2012 [4]. Optimal patient care requires the development of laboratory diagnosis of infections and knowledge on the local epidemiology of infections, both of which are currently limited. We conducted the first laboratory-documented study on endemic and opportunistic intestinal parasites in HIV-infected patients, with or without diarrhea. In particular, we used for the first time in Laos specific methods for the detection of microsporidia, *Cryptosporidium* and other coccidia in stool samples.

### Clinical and immunological status of the population

This cross-sectional study was carried out in the first two implemented and most important ART centers of the country, one located in the capital Vientiane (Setthathirath Hospital) and the other in a Southern province (Savannakhet Hospital). In this population, HIV infection was diagnosed in young (median age: 36 years) and severely immunocompromised patients, as assessed by WHO clinical staging criteria (83.9% of patients classified as stage 3 or 4) and low median CD4 cell count (41 cells/mm^3^). Three factors may explain the late diagnosis of HIV infection. Firstly, the majority of patients (54.0%) lived in rural areas, especially in Southern provinces where information about HIV infection may not be widespread. Secondly, HIV screening tests are not available in all the district hospitals and health centers, which means that patients are required to travel to the diagnostic centers. Finally, Lao people use traditional medicine first and go to hospitals only if traditional methods fail [18]. Our results support previous data on severely immunocompromised patients at HIV diagnosis in Laos [3], [4] and confirm the urgent need for suitable care of patients who are at high risk of opportunistic infections. The low prevalence of pulmonary tuberculosis and cryptococcal meningitis reported here (7.3% and 8.0% respectively) is most likely an underestimation. Due to the low diagnostic rate of the microbiological tools routinely used in these hospitals and their limited availability (less than 40% of the patients underwent laboratory investigations for opportunistic infections), it is very likely that some diagnoses were missed.

### Diarrhea and parasite infections

Nearly half of the patients presented with diarrhea, of which one third presented with persistent diarrhea of more than two weeks duration. This proportion of diarrheal HIV patients is closer to that reported in industrialized countries before the highly active antiretroviral therapy era (40 to 80%) [19] than to the proportion that is usually observed in developing countries (up to 95%) [20], [21]. This may be related to under-reporting of diarrhea by physicians or by the patients themselves. The clinical picture was more severe and compatible with more advanced WHO stages of HIV infection in diarrheal patients compared to non-diarrheal ones. However, we found no correlation between diarrhea and immunological failure as assessed by CD4 cell counts. No demographic characteristic or environmental condition (place of residence, living conditions) was found to be associated with diarrhea, except living in the Southern provinces. This might be due to the fact that our study sample was relatively small and therefore, small risk differences could not be detected.

Parasitological analyses of stool specimens revealed high prevalence rates of parasitism (78.8%), multiparasitism (presence of at least two different parasite species: 49.6%), protozoan infection (54.7%) and helminth infection (58.4%). Such findings in HIV-infected patients were not reported from neighboring countries in Southeast Asia [22], [23], [24], [25], [26], [27], [28], [29]. Comparing prevalence rates of parasitic infections in HIV-infected patients amongst different countries or regions requires caution. The observed variations may be due to the different study designs and laboratory techniques used. On the other hand, they may reflect true differences in epidemiological features and specific risk factors from each country or region. Concerning the high prevalence of parasitism, multiparasitism and helminth infections, our results from HIV patients are in agreement with those of previous studies on the general population of Laos [6], [7], [9], [10], [11].

We investigated the association between intestinal infection,CD4 cell count and diarrhea. Compared to patients with CD4 cell count >50 cells/mm^3^, patients with CD4 count ≤ 50 cells/mm^3^ were more likely to be affected by intestinal parasitism (with any parasite, protozoa or helminth or *O. viverrini*) and multiparasitism with more than two species. Persistent diarrhea and cryptosporidiosis was the unique association observed between diarrhea and parasite infection.

### Protozoa infections

With the Kato smear method being the unique diagnostic technique, protozoan infections are under-diagnosed in Laos. Virtually all results reported from the general population concern helminth infections [6], [7], [9], [10], [11]. Considering the recent emergence of HIV infection, improving biological diagnosis and knowledge about intestinal protozoan infections is crucial. We found that protozoan infections, affecting half of the population, were almost as prevalent as helminth infections. *Cryptosporidium* sp. and microsporidia are described here for the first time in Laos, with low prevalence rates of 6.6% and 2.9% respectively. The prevalence rates of cryptosporidiosis and microsporidiosis in HIV-infected population varied widely in previous studies from industrialized and developing countries, including neighboring countries of Laos. Prevalence of cryptosporidiosis varied from 0 to 100% with a median of 32%, and that of microsporidiosis from 5 to 50% with an overall prevalence of 15% [22], [23], [24], [25], [26], [27], [28], [29], [30], [31], [32], [33], [34], [35], [36], [37], [38]. The presence or absence of diarrhea was not always specified in these studies. In Lao patients, persistent diarrhea was clearly linked to cryptosporidiosis as already reported in HIV-infected patients from various countries around the world [32], [34], [37]. Other opportunistic coccidia, *Cytoisospora belli* and *Cyclospora cayetanensis*, were found in our patients, with or without diarrhea. To our knowledge, *C. belli* has not been described before in Laos. To date, *C. cayetanensis* was mentioned in only one study, in which it was found to be responsible for 0.1% of diarrheal cases [39]. HIV status of the patient was not specified. The prevalence rates of *C. belli* and *C. cayetanensis* infections in our patients (4.4% and 2.2%, respectively) were close to those reported in HIV-infected patients in two neighboring countries, Thailand and Cambodia [22], [24], [25], [26], [28], [35], [40]. Studies on coccidia and microsporidia infections should be performed on the general population of Laos to confirm their low prevalence rates and to investigate their specific epidemiological features.

A striking finding in this study was the high frequency of the emergent pathogen, *Blastocystis* (26.3%). A high prevalence of *Blastocystis* in HIV-positive patients was also reported in China (19.2%) [27], contrary to Thailand where a prevalence of approximately 2% was found [23], [25], [26], [28]. *Blastocystis* was recently described for the first time in Laos, with a prevalence of 13.6% in the general population of the Champasack province [9]. *Blastocystis* appeared to be the most prevalent protozoa both in general and in HIV populations from Laos, but the rate of *Blastocystis* carriage seemed to be higher in HIV-positive patients, a finding already reported elsewhere [37], [41]. Clinical features were not specified in the participants of the Champasack study [9]. No association between *Blastocystis* and diarrhea or other digestive symptoms was found in our HIV patients, as reported in many previous studies in immunocompetent or immunocompromised patients [37], [41]. The role of *Blastocystis* in gastrointestinal disease, which may be related to specific subtypes and parasite burden [42], [43], [44], is still controversial. However, it seems currently wise to consider *Blastocystis* as a pathogen in immunocompromised persons, such as HIV-infected patients, and to treat the infection [37], [41].

Data from Laos on *G. intestinalis* and *E. histolytica/dispar* infections as for other protozoan infections, is scarce. Their prevalence rates in the general population seemed lower than those in the HIV-positive population : less than 0.5% [9], [45] *vs*. 12.4% for *E. histolytica/dispar* infection, and around 4.5% [8], [9] *vs*. 8.8% for *G. intestinalis* infection. Neither acute nor persistent diarrhea was associated in our study with *E. histolytica/dispar* or *G. intestinalis* carriage. In fact, none of these two infections is currently considered as opportunistic, even though HIV infection was reported to increase the risk of developing invasive amoebiasis and symptomatic *G. intestinalis* infection with progressive immunosuppression following reduced CD4 cell counts [37], [46].

### Helminth infections

As expected, helminth infections were highly frequent in the Lao HIV-positive population. Living in the Southern provinces appeared to be associated with helminth infections, which were also associated with the absence of in-house toilets. Inadequate sanitation, recognized as a determinant factor for parasitic infections, was reported to be a major problem in Laos [9]. Such a frequency of HIV-helminth co-infection and moreover, of HIV-multiparasitism association, requires particular attention in Lao patients. The immune modulation by helminths combined with the progressive immunodeficiency by HIV may have deleterious effects on both HIV acquisition and disease progression and on increased susceptibility to parasitic infections [27], [47], [48].

No helminth species was associated with diarrhea in the HIV-infected patients of the study, even *S. stercoralis*. Strongyloidiasis was associated with living in rural areas and was more frequent in Southern provinces than in Northern provinces. The overall prevalence was high (20.4%). Lower prevalence rates were found in the general population from Laos [6], [7], [8], [9], [10], [11] as well as in HIV-infected patients from neighboring countries [22], [24], [25], [27], [28], [29], [49]. With the increasing use of antiretroviral drugs in developing countries, the problem of possible immune reconstitution inflammatory syndrome (IRIS) to *S. stercoralis* infection has recently emerged [50], [51]. Considering the high prevalence of strongyloidiasis and the frequent advanced stage of immunosuppression at HIV infection diagnosis in Laos, the risk of developing *Strongyloides* IRIS should be carefully assessed and prevented when initiating ART.

Small flukes were the most prevalent helminths in the HIV population (47.5%), as already reported in the general population [6], [7], [10]. *O.viverrini* eggs and those of minute intestinal flukes belonging to the Heterophyidae, Lecithodendriidae and Echinostomatidae families were not differentiated. This procedure requires adult worm recovery or molecular identification [6], [7], [10], [52]. Since the highest *O. viverrini* prevalence rates have been reported from Southern provinces along the Mekong River [52], [53], we can postulate that our samples originating from Bolikhmaxay, Savannakhet, Khammuane, Champasack and Saravane provinces, were mainly infected with *O. viverrini*. Opisthorchiasis is recognized as a major health problem in Laos because of high prevalence and associated chronic hepatobiliary abnormalities and cholangiocarcinoma [52], [53]. The estimated prevalence of cholangiocarcinoma in people living in endemic areas was 5% [52], [53]. The high frequency of HIV – *O. viverrini* co-infection raises the question of a potentially increasing occurrence of cholangiocarcinoma in surviving Lao AIDS patients treated with ART. The increased risk of non-AIDS-defining cancer in HIV-infected persons compared to the general population has already been pointed out in developed countries in the ART era [54], [55]. The occurrence of several other cancer types might be due to the combination of HIV-induced immunosuppression and oncogenic infection [56], [57]. *O. viverrini* is considered as a type 1 carcinogen by the International Agency for Research on Cancer, World Health Organization [53].

### Limitations of the study

There were some limitations to this study. Firstly, the possibility that the patients had treated themselves with antibiotics or antiparasitic treatments prior to the hospitalization could not be precluded: sulfamethoxazole-trimethoprime, metronidazole, mebendazole, albendazole and praziquantel are available without prescription in Laos. Secondly, 23.3% of the patients gave three stool samples: the analysis of only one or two stool samples might lead to missed parasitological diagnoses. Thirdly, the techniques used have a relatively low sensitivity for the detection of some parasite species and might also have led to a certain underdiagnosis.

## Conclusions

Parasitism and multiparasitism were common in patients at HIV diagnosis in Lao PDR, as previously reported in the general population. Novel features were found in the HIV-infected patients, with or without diarrhea. Firstly, protozoa infections were almost as prevalent as helminth infections, the more frequent being *Blastocystis* sp. infection. Secondly, coccidia (*Cryptosporidium* sp., *C. belli*, *C. cayetanensis*) and microsporidia were observed, and *Cryptosporidium* sp. was associated with persistent diarrhea. Finally, *S. stercoralis* was the most prevalent helminth following *O. viverrini*. The most immunocompromised patients, as assessed by a CD4 count ≤ 50 cells/mm^3^, were more likely to be infected with parasites, protozoa, helminths and *O. viverrini*. Since HIV infection is currently diagnosed at an advanced stage of immunosuppression in Laos, our findings highlight the importance of laboratory diagnosis of intestinal parasitic infections including protozoa, and the need for adequate care and treatment. Further studies are required: i) to assess the prevalence and specific risk factors of intestinal protozoan infections in the general population of Laos, ii) to genetically characterize protozoan isolates, iii) to confirm and complete our clinical and epidemiological data on parasitic infections in HIV-positive patients from all the centers of the country and to search for bacteria and viruses in the stools of diarrheal patients.
